# Numerical Study of Fabrication-Related Effects of the Structural-Profile on the Performance of a Dielectric Photonic Crystal-Based Fluid Sensor

**DOI:** 10.3390/ma15093277

**Published:** 2022-05-03

**Authors:** Yousuf Khan, Muhammad A. Butt, Nikolay L. Kazanskiy, Svetlana N. Khonina

**Affiliations:** 1 Technological Electronics, Institute of Nanostructure Technologies and Analytics, University of Kassel, Heinrich-Plett-Str.40, 34132 Kassel, Germany; 2 Nanophotonics Research Group, Department of Electronic Engineering, Balochistan University of Information Technology, Engineering and Management Sciences, Quetta 87300, Pakistan; 3 Samara National Research University, 443086 Samara, Russia; 4 Institute of Microelectronics and Optoelectronics, Warsaw University of Technology, Koszykowa 75, 00-662 Warszawa, Poland; ali.butt@pw.edu.pl; 5 IPSI RAS-Branch of the FSRC “Crystallography and Photonics” RAS, 443001 Samara, Russia; kazanskiy@ipsiras.ru (N.L.K.); khonina@ipsiras.ru (S.N.K.)

**Keywords:** dielectric photonic crystals, low-cost biosensors, focused ion-beam technology, structural profile, guided-mode resonance

## Abstract

In this work, fabrication of a dielectric photonic crystal device and numerical study of its spectral characteristics as a refractive index sensor are presented for near infrared range. The proposed nanosensor device is composed of low-cost dielectric materials, i.e., silicon dioxide and niobium pentoxide, and is fabricated using focused ion-beam milling lithography. In the first part, the fabrication process of the device is discussed, along with the process parameters and their effects on the structural properties of the resulting photonic crystal elements. In the second part, the device is numerically tested as a sensor for the biological refractive index range of 1.33 to 1.4. The performance considerations of the biosensor device are studied for 12 different structural profiles based on the fabrication results. It is shown that the angular-wall-profile of the fabricated structures downgrades the performance of the sensor, and the optimum value of hole depth should be in the range of 930–1500 nm to get the best performance. A sensitivity of 185.117 nm/RIU and a figure of merit of 9.7 were recorded for the optimum design of the device; however, a maximum sensitivity of 296.183 nm/RIU and a figure-of-merit of 13.184 RIU^−1^ were achieved. The device is recommended for a variety of biosensing applications due to its inert material properties, stable design and easy integration with fiber-optic setups.

## 1. Introduction

As modern-day technology is moving towards compact, fast and lab-on-chip devices, demand for miniaturized, ultra-power-efficient and non-destructive sensing techniques is rising. Considering the above-mentioned merits, optical sensing and spectroscopy have already been adopted in many opto-electro sensing applications. All-optical sensing techniques have long been researched with the goal of exploring new meta-materials, spectral ranges and application areas. Among nanostructures, photonic crystals (PhCs) have proven to be one of the favorite candidates for sensing applications, due to their ability to manipulate and filter light at a wavelength scale. Recently, the application of PhCs in sensing has been a primary topic of research, and they have been used in gas sensing [1,2,3], plasmonic biosensing [4,5], refractive index sensing [5,6,7,8,9], bacteria sensing [10,11,12], temperature sensing [13], bimolecular sensors [14] and other applications. As compared to their semiconductor counterparts, dielectric material-based PhCs offer very low absorption over a wide spectral range from visible to near-infrared (NIR), which makes them suitable for a wide range of applications [15,16]. Moreover, due to the low-cost of dielectric materials, inert chemical properties and easier integration with optical fiber-based setups, dielectric PhCs devices offer overall cost-effective fabrication, making them suitable for sensing applications. Common fabrication technologies for complex periodic structures, such as PhCs, include electron-beam (E-beam) lithography [17], reactive-ion etching (RIE) [18], focused ion-beam (FIB) technology [19], nano-imprint lithography [20] and material processing using high energy fs-laser pulses [21]. FIB technology is considered one of the accurate and swift prototyping techniques for PhCs devices, since it offers various tunable constraints, making the fabrication and characterization processes versatile. An overview of PhC-based sensors is reported in [6]. Recently, refractive index sensing of fluids using PhC structures has been presented in [7,8,9,22,23,24,25,26]. Design and fabrication of sensors for refractive index sensing of liquids using PhC fibers are reported in [25,26,27]. Moreover, 1D and 2D PhC micro-cavities are reported for index sensing in [28,29,30,31]. Selected works have reported on pure dielectric PhC structures for biosensing applications [32,33,34]. Fabrication of nanophotonic devices by direct ion-beam lithography [35,36,37,38,39,40,41] and challenges faced due to FIB irradiation, such as structural deformations [36,37,38,39], ion-beam erosion [37], redeposition of particles [37,38,39], material swelling [37,39], side-wall angles [37,39] in nanostructures and surface charging of dielectric materials [40], have also been reported. However, a detailed analysis of the effects of the fabrication-related structural profiles of dielectric materials on the performance of a sensing device has not been yet reported.

This work focused on fabrication, numerical modeling and testing of dielectric PhC-based nanosensor devices for fluid sensing applications in the NIR spectral range. Considering their wide spectral range, low absorption, material cost and easy prototyping, two commonly available dielectric materials, i.e., silicon dioxide (SiO_2_) and niobium pentoxide (Nb_2_O_5_) were chosen for design of the presented nanosensor device. The discussed PhC structures work on the principle of guided-mode resonance (GMR), also known as Fano-resonance [19,42]. GMR works on the principle of out-of-the-plane coupling of light into the structures, where the free space modes interfere with the guided modes inside the structures with a phase-matching mechanism to create resonances. Moreover, to enable rapid prototyping, structural characterization and SEM imaging in one platform, an FIB milling lithography-based fabrication technique was chosen. The deformation and variations in the structural properties of the sensor device due to FIB process parameters are reported. The proposed device was numerically investigated as a fluid sensor for the biological refractive index range of 1.33–1.40. The effect of structural deformation on the performance of the fluid sensor was studied for 12 different structural designs of PhC elements. The investigated spectral properties included variations in resonant wavelengths, the linewidth of Fano resonances, the sensitivity (S) of the device and the figure of merit (FOM). The performance of the device is compared with previously reported work on index sensing using similar materials and refractive index ranges.

## 2. Fabrication of the PhC Nanosensor Device

**Figure 1:**
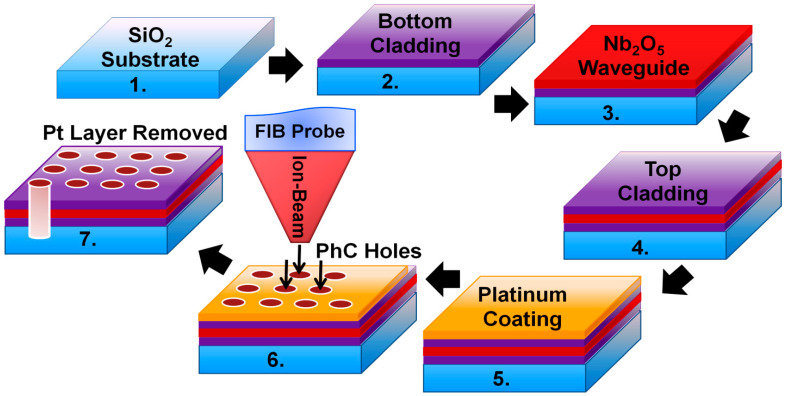
Experimental procedure for the fabrication of a dielectric PhC nanosensor device.

Fabrication of the dielectric PhC nanosensor device includes two main steps, i.e., deposition of the thin-films and structuring of the air holes. A detailed overview of the fabrication process is given in Figure 1. An all-solid-layer device model was chosen instead of suspended membranes to achieve a mechanically stable and easy to fabricate device model. The thin-film layers were deposited on a borosilicate glass substrate using the ion-beam sputter deposition (IBSD) method (Figure 1, steps 1 to 4). The deposited layers consist of a bottom cladding layer of SiO_2_, an Nb_2_O_5_-based waveguide layer and a top cladding layer of SiO_2_. The Nb_2_O_5_ layer s submerged between two SiO_2_ cladding layers to achieve a symmetric waveguide design. The two SiO_2_ cladding layers have a thickness of 300 nm, and the Nb_2_O_5_ layer is 330 nm thick. The layer thicknesses were decided as per the optimized simulation model of the device. A dual beam IBSD machine *IonSys* 1000 from *Roth&Rau* (Hohenstein-Ernstthal, Germany) was used for deposition. The primary ion beam sputters the desired materials, i.e., Si and niobium from the target maintaining a constant flux with a typical vacuum condition of 1.7 × 10^−7^ mbar inside the chamber. The substrate holder is placed at a distance of 30 cm from the target with a tilt angle of 45° and rotates at a rate of 30 rpm. A secondary ion source directs a plasma of oxygen and argon gas towards the substrate to enable deposition of SiO_2_ and Nb_2_O_5_. Primary ion beam currents of 72 and 75 mA were used for deposition of SiO_2_ and Nb_2_O_5_, respectively, with an acceleration voltage of 100 V. A secondary ion beam current of 10 mA was adopted for deposition of both SiO_2_ and Nb_2_O_5_. The duty cycle of the primary ion source was set to 45% for SiO_2_ and 72% for Nb_2_O_5,_ whereas for the secondary source it was 17% for SiO_2_ and 13% for Nb_2_O_5_. The deposition rates of SiO_2_ and Nb_2_O_5_ were measured to be 3.54 and 1.68 nm/min, respectively. Since the device was designed to work in the NIR range, the refractive indices of the deposited thin-films were measured to be n=2.2 for Nb_2_O_5_ and n=1.5 for SiO_2_ layers [19,21]. A 10 nm platinum (Pt) layer (Figure 1, step 5) was deposited on the top of the specimen to drain the static charge accumulated on the dielectric surface during scanning electron microscopy (SEM) imaging and FIB milling. A cross-beam dual platform FIB/SEM machine *NVision 40* manufactured by *Carl Zeiss SMT* (Oberkochen, Germany) was used for structuring of PhCs. The chamber is maintained at a vacuum condition of below 10^−6^ mbar and the substrate holder is oriented at an angle of 54° to make it perpendicular to the plane of ion-beam during the milling process. When the specimen is placed into the vacuum chamber of the FIB machine, the surface of the specimen is ground with the mounting stage to improve the accuracy of the process. As per the optimized numerical simulation model, a software-based mask was designed for lithography on the standard GDSII compatible software provided by *Raith Elphy* [36,41]. As the fabricated sensor device was targeted for operation in NIR spectral range, the lattice constant of the periodic structure was designed to be *a* = 1 µm. During the milling process, the deflection of the ion beam (Figure 1, step 6) is controlled following the provided software design. The milling process can be monitored by the live-milling mechanism provided by the FIB machine, which allows SEM visualization of the process.

To reach an optimized fabrication recipe, several experiments were performed where mainly the ion-beam current, area dose and number of process loops were varied. The primary goal was to achieve a structural profile of the milled holes as close to the numerical design as possible. Choosing the ion-beam current value is a trade-off between the quality of the structures and the total process duration; i.e., higher currents mill faster but the quality of the is structures decreased and vice versa. It is important to mention that the experimental values of the implemented structures also largely depend on the nature of the subject material. Dielectric materials are comparably hard to mill and take a longer process duration as compared to their semiconductor counterparts.

**Figure 2:**
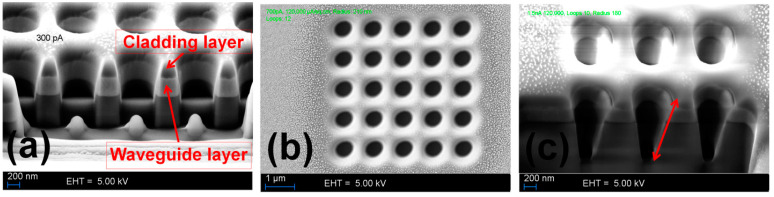
SEM images of fabricated dielectric PhC structures. (**a**) Cross-sectional view of a PhC structure milled with an ion-beam current of 300 pA. Holes reach out equally on both sides of the waveguide layer. (**b**) Top view of a 5 × 5 grid of PhC holes milled with an ion-beam current of 300 pA. (**c**) PhC structures milled with an ion-beam current of 1500 pA. The holes posses a conical structural profile indicated with a red arrow and are approximately 1.5 µm deep.

The cross-sectional and top view of the milled PhC elements are shown in Figure 2a–c. To achieve a perfectly cylindrical wall profile of the milled holes, an ion-beam current in a range of 80 pA must be used which would extend the process duration exponentially. Therefore, the quality of the milled structure was investigated for ion-beam currents in the range of 300 to 1500 pA for an area dose value of 120,000 μAs/cm^2^. The milling process must also be divided into multiple process loops to achieve the desired structural depth. By observing the cross-sectional views of different milled structures in Figure 2a,c, it can be seen that as the ion-beam current was varied from 300 to 1500 pA while keeping the area dose and process loops constant. The depth and shape of milled air holes were also changed. The air holes milled at lower beam current were less deep and had a smoother and steep wall profile (Figure 2a). However, as the beam current was increased, the shape of the air holes became more conical, leaving an angled wall profile and higher aspect ratio (Figure 2c). To give a detailed account of variations in the structural profiles of the milled PhC elements at different ion-beam currents, the numerical values of side-wall-angles averaged over several readings for each ion-beam current are listed in Table 1. After generating the cross-sectional views of the milled structures, the side-wall-angles were measured separately for the left and the right walls during SEM imaging to make the readings more authentic. The standard error of the mean was calculated from the standard deviation of the averaged side-wall-angle values. Considering the listed values in Table 1, it can be noticed that the ion-beam current of 300 pA offers a side-wall-angle near 12°, whereas for 700 pA it is around 16° and for 1500 pA it is around 14°. It can be observed that the angular-deviations are highest at 700 pA rather than at the highest ion-beam current of 1500 pA. The reason can be explained in terms of material properties and spot size of the ion-beam. The numerical models of the PhC elements were generated by carefully observing the structural properties of the fabricated structures. In the final step of fabrication, the Pt layer was removed by wet etching, as it could scatter the incident light during the optical characterization and testing of the nanosensor device.

## 3. Numerical Simulation Method

**Figure 3:**
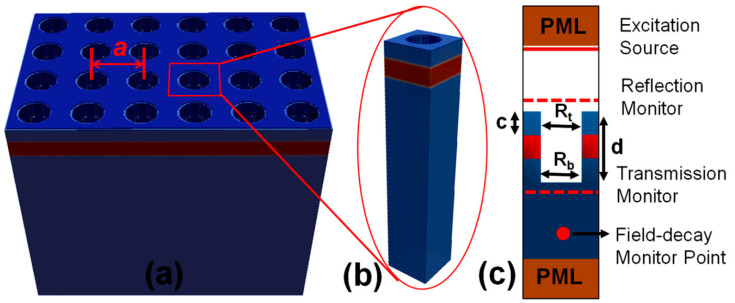
Simulation model of the fabricated dielectric PhC device. (**a**) A 3D model of the device with a waveguide layer on top of the substrate and covered by a cladding layer. (**b**) 3D view of the unit cell model used in the simulation. (**c**) Cross-sectional view of the unit cell model showing PhC structure, source, field monitoring points and boundary conditions.

The numerical designing and simulation of the nanosensor device were performed in open-source finite-difference time-domain (FDTD)-based simulation software called MIT electromagnetic equation propagation (MEEP) [43]. The numerical model of the fabricated device is shown in Figure 3a, where PhC elements are arranged in a square lattice and the lattice constant of the device is indicated as a. The unit-cell modeling technique was used to enable several computations of the model, saving time and computational resources. A 3D model of the unit cell design is shown in Figure 3b depicting a waveguide layer submerged between a substrate and cladding layer with an air hole as the PhC element. A cross-sectional view of a unit cell model is shown in Figure 3c showing PhC structure, the position of the excitation source, reflection and transmission flux monitor layers and the decay monitor point. The structural parameters, such as the upper radius of the PhC structure $R_{t}$, bottom radius $R_{b}$, depth d and cladding layer thickness c, are also indicted. The field decay monitor point (Figure 3c) basically checks magnitude of the oscillating field to decide when to stop the simulation as per user-defined criteria.

A perfectly matched layer (PML) boundary condition is used in z-direction (top and bottom) to absorb the outgoing field and avoid back-reflections. Periodic boundary conditions (PBCs) were applied in (x and y) directions to simulate a perfect crystal structure. Since the designed device can also be scaled to operate in other spectral ranges, the thickness of the waveguide and radius of the hole are expressed in terms of lattice constant. Correspondingly, the cladding layer thickness of 300 nm can be expressed as c=0.30a and a standard air hole radius of R=0.30a. To graphically visualize the results, the time domain simulation outcomes are transformed to the frequency domain by Fourier transformation.

## 4. Sensing Properties of the Dielectric PhC Device

**Figure 4:**
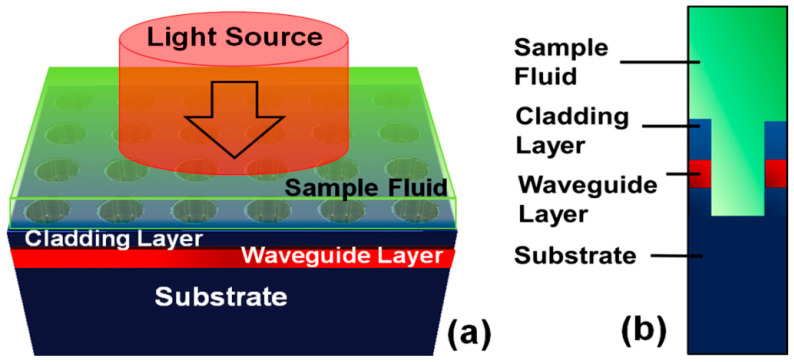
Numerical model of the nanosensor device. (**a**) Full 3D model of the device with the presence of a layer of the sample fluid on top of the PhC structure and an incident light beam. (**b**) Cross-sectional view of the unit cell model showing PhC structure and presence of the sample fluid.

The sensing capabilities of the fabricated nanosensor device may deviate from the standard theoretical model due to modifications in the structural and material properties of the PhC structures during FIB processing [36,37,38]. Therefore, the spectral response of the sensor device was investigated for various structural properties resulting from the fabrication process, i.e., the shape of the PhC elements, their depth and the thickness of the cladding layer. The sensing properties were numerically investigated by changing the ambient refractive index $n_{a}$ in the biological refractive index range from $n_{a}=1.33$ to 1.40. For visual clarification, a full 3D model of the sensor device with the presence of sample biological fluid (light green color) and an incident light source is depicted in Figure 4a. Moreover, Figure 4b shows a cross-sectional view of the unit cell model showing the presence of sample fluid above the PhC structure.

**Figure 5:**
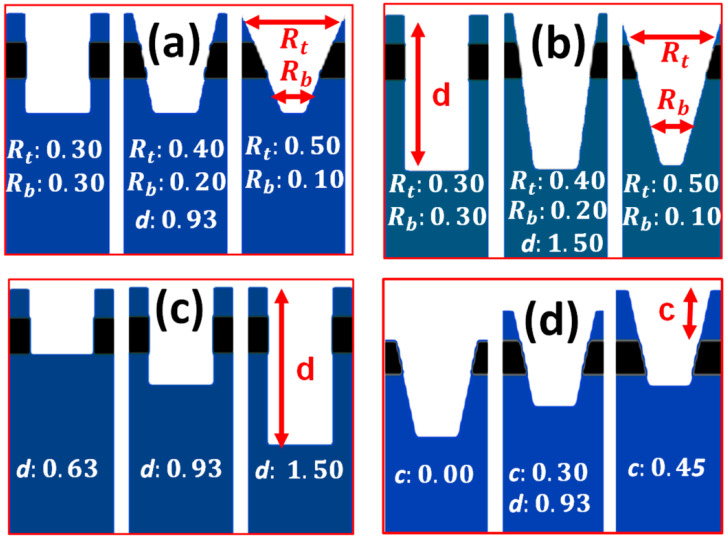
Different structural profiles of the PhC elements are considered for performance evaluation of the fabricated nanosensor device. (**a**) Variation in the shape of PhC elements with d=0.93a. (**b**) Variation in the shape of PhC elements for deeper holes with d=1.50a. (**c**) Variation in the depth of PhC elements for cylindrical hole shape. (**d**) Variation in the thickness of cladding layer for PhC element shape of $R_{t}/R_{b}=0.40/0.20a$ at depth of d=0.93a.

The performance characterization of the device for sensing of biological fluids was assessed by calculating the variations in resonant wavelengths $\lambda_{res}$, the linewidth of the resonant modes, S and FOM of the nanosensor device. Moreover, the variations in the sensing performance of the device for different structural properties resulting from FIB processing were evaluated by comparing the spectral response and sensitivity values. The S and FOM of the sensor device are given by Equations (1) and (2). The S of the device is expressed as nm per refractive index unit (nm/RIU) and FOM is expressed in RIU^−1^.

(1) $$S=\frac{\Delta \lambda_{res}}{\Delta n_{a}}$$

(2) $$FOM=\frac{S}{\mathrm{Linewidth}}$$

where $\Delta \lambda_{res}$ is the shift in $\lambda_{res}$ for a change in the refractive index $\Delta n_{a}$ of the sample fluid. Figure 5 depicts the numerical models of different structural profiles of the PhC elements resulting from the FIB material processing. Figure 5a,b shows change in the shape of PhC element at two different hole depths, i.e., d=0.93a and d=1.5a. The shape varied from cylindrical holes to conical holes by changing the values of $R_{t}$ and $R_{b}$. Figure 5c shows variation in the hole depth of the PhC elements in a standard cylindrical hole model with $R_{t}/R_{b}=0.30/0.30a$. Figure 5d shows variation in the thickness of the cladding layer with a PhC element shape of $R_{t}/R_{b}=0.40/0.20a$. A detailed study of the effects of above-mentioned structural parameters on the performance of the sensor device is given in the upcoming sections.

### 4.1. Shape of PhC Elements

**Figure 6:**
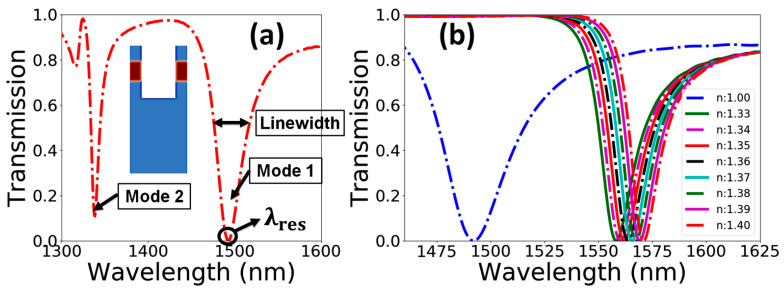
(**a**) Transmission and reflection spectra of the standard PhC structure with cylindrical air holes with $R_{t}/R_{b}=0.30/0.30a$. (**b**) Redshift in $\lambda_{res}$ as the ambient index varies from $n_{a}=1.33$ to 1.40 in a sensor device with cylinderical air holes. Dotted blue line indicating the position of resonance with air as the ambient medium.

As per the standard numerical model, the patterned air holes should be cylindrical. However, the fabricated structures may possess angular wall profiles following the material properties and the ion-beam currents used in prototyping. The shape of the PhC element affects the quality of Fano-resonances and the location of $\lambda_{res}$ in the spectral range. These performance considerations become more crucial while using these PhC structures for sensing applications. For instance, in the case of a refractive index sensor presented in this work, the shifting in the $\lambda_{res}$ in output spectra determines the physical properties of the sample fluid, but $\lambda_{res}$ may also shift due to modifications in the structural properties of the PhC elements. Therefore, the performance of the device needs to be investigated for all the structural features resulting from the fabrication process. Figure 6a shows the transmission and reflection spectra of a standard cylindrical-wall-profile dielectric PhC sensor device with $R_{t}/R_{b}=0.30/0.30a$ and a standard hole depth of d=0.93a. It can be noticed that the device has two sharp resonant peaks in the studied spectral range of around 1350 and 1490 nm. Both peaks can be used for a comprehensive sensing response. However, this work only considers one resonant peak located around 1490 nm for sake of simplicity. Figure 6b plots $\lambda_{res}$ for the device without the presence of a sample fluid and the shifting of $\lambda_{res}$ during sensing of biological fluids. The dotted blue line shows the spectral response of the device for an ambient refractive index of $n_{a}=1.0$ (referring to air), and the rest of the resonant peaks represent the spectral resonance of the device for the biological refractive index range of $n_{a}=1.33\;\mathrm{to}\;1.40$ with a step size of 0.01. The sensor device shows a linear redshift in $\lambda_{res}$ as the $n_{a}$ increases. Similalry, the sensing response of the device was computed for PhC element profiles of $R_{t}/R_{b}=0.40/0.20a$ and $R_{t}/R_{b}=0.50/0.10a$ at two different hole depths of d=0.93a and d=1.50a.

**Figure 7:**
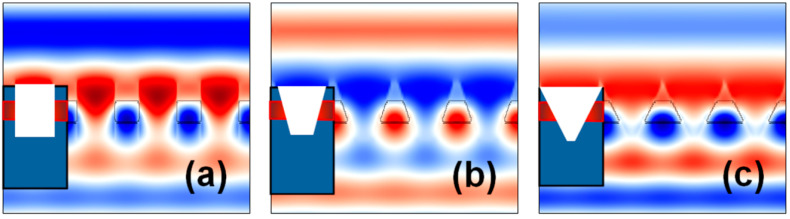
EM field distributions in the PhC structures where the maxima and minima of the EM field are represented by red and blue colors, respectively. (**a**) $R_{t}/R_{b}=0.30/0.30a$. (**b**) $R_{t}/R_{b}=0.40/0.20a$. (**c**) $R_{t}/R_{b}=0.50/0.10a$.

The electromagnetic (EM) field distributions in the PhC structures during the occurrence of Fano resonance in relation to the change in the PhC element shape from cylindrical to conical is shown in Figure 7. It can be shown that the cylindrical structural profile offers better localization of the resonant mode as compared to the conical hole shape, since it offers a symmetric waveguide design.

**Figure 8:**
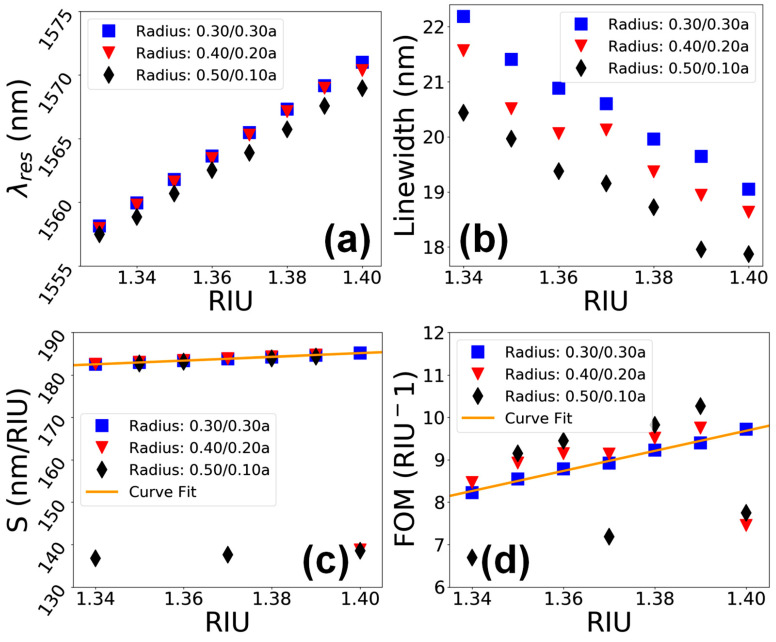
Performance evaluation of the sensor device vs. variation in the shape of PhC elements for a hole depth of d=0.93a. (**a**) $\lambda_{res}$ vs. RIU. (**b**) Linewidth vs. RIU. (**c**) S vs. RIU. (**d**) FOM vs. RIU.

A detailed analysis of the sensing performance of the device in terms of $\lambda_{res}$, linewidth, S and FOM for the three different hole shapes, i.e., $R_{t}/R_{b}=0.30/0.30a$, 0.40/0.20a and 0.50/0.10a, at a standard hole depth of d=0.93a, is given in Figure 8. Figure 8a shows that the $\lambda_{res}$ undergoes a redshift in range of $\lambda_{res}=1555\;to\;1570$ nm in all the hole shapes as the value of $n_{a}$ increases. Figure 8b depicts that the linewidth of resonant peaks has an inverse relation with the RIU, and the linewidth reduces almost linearly from 22 to 18 nm in the case of all three considered PhC element shapes. The S of the device as a function of RIU in Figure 8**c** shows that the hole shapes with $R_{t}/R_{b}=0.30/0.30a$ and 0.40/0.20a offer linear variation in value of S in range of 180 to 190 nm/RIU. However, when the shape of PhC elements becomes more conical, the value of S fluctuates over a wide range of values between 135 to 185 nm, as shown in the figure. Similarly, the FOM of the nanosensor device (Figure 8d) shows that the hole shape values of $R_{t}/R_{b}=0.30/0.30a$ and 0.40/0.20a relate to a linear change in FOM in the range of 8 to 10 RIU^−1^ as the values of $n_{a}$ vary. In the case of the extreme conical shape of PhC elements, the FOM fluctuates between 6 and 10 RIU^−1^.

**Figure 9:**
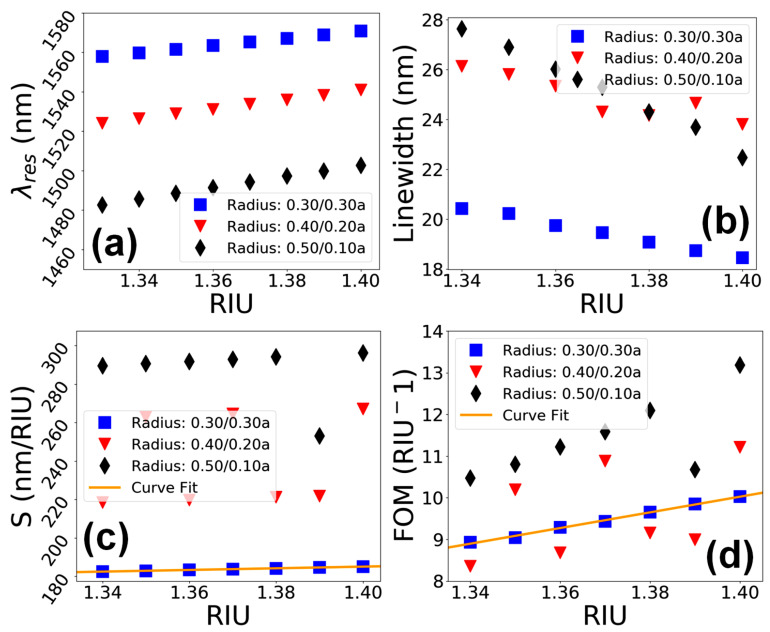
Performance evaluation of the sensor device vs. variation in the shape of PhC elements for hole depth of d=1.5a. (**a**) $\lambda_{res}$ vs. RIU. (**b**) Linewidth vs. RIU. (**c**) S vs. RIU. (**d**) FOM vs. RIU.

Practically, it is almost impossible to achieve a straight wall profile for PhC elements deeper than 1 µm using FIB technology and working with dielectric materials. Therefore, the performance of the sensor device was also investigated for deeper PhC holes of d=1.5a. Since the shape of PhC elements vary with the value of ion-beam current used for milling, a whole range of PhC element shapes, i.e., $R_{t}/R_{b}=0.30/0.30a$, 0.40/0.20a and 0.50/0.10a, were considered for the performance evaluation of the sensor device. Figure 9a shows that $\lambda_{res}$ varies linearly for all the PhC elements’ shapes, and $\lambda_{res}$ undergoing a slight redshift as the shape of the holes transforms from cylindrical to conical. The linewidth plot in Figure 9b depicts a linear modification for all the hole shapes over a value range of 18 to 28 nm. Considering the trends for S of the device in Figure 9c, it can be observed that the device performs linearly, reaching 185 nm/RIU, for steep-walled and less conical hole profiles. In extremely conical hole shapes, S varies non-linearly, reaching 290 nm/RIU, as the value of $n_{a}$ increases. Lastly, considering the FOM of the device in Figure 9d, a linear behavior in range of 9 RIU^−1^ can been seen for the straight wall profile of the air holes, and it turns out to be non-linear as the shape of the holes becomes conical.

### 4.2. Depth of PhC Elements

**Figure 10:**
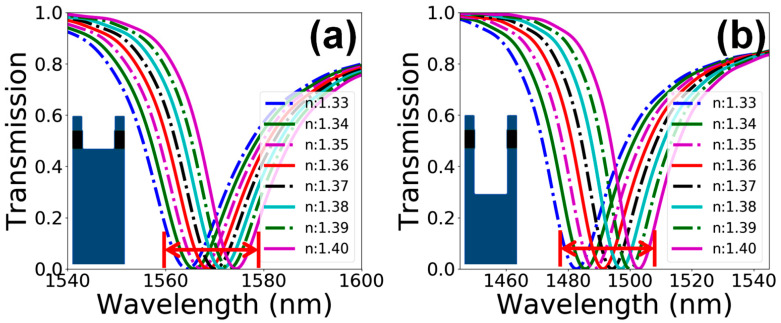
Transmission spectra of a dielectric nanosensor device vs. variation in the ambient refractive index value. (**a**) Hole depth d=0.63a. (**b**) Hole depth d=1.5a.

**Figure 11:**
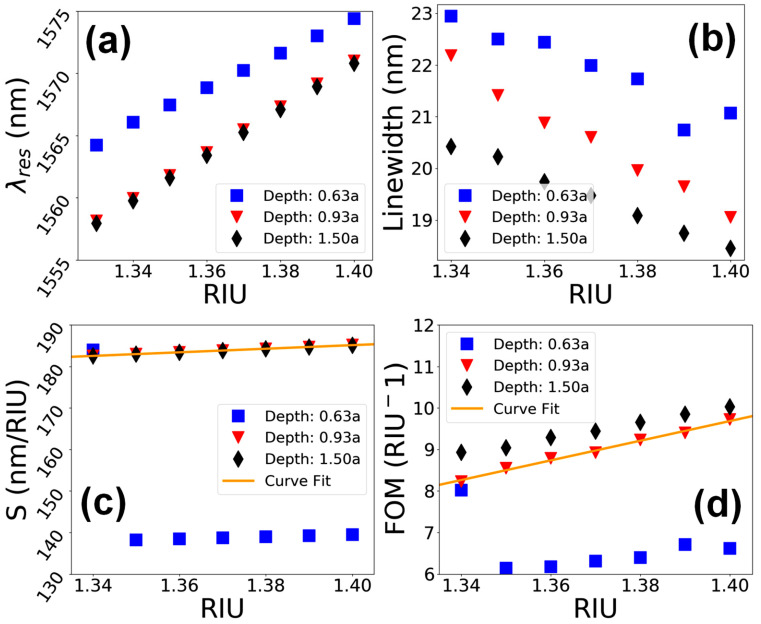
Performance evaluation of the device for hole depths of d=0.63a, 0.93a and 1.5a in cylindrical PhC elements. (**a**) $\lambda_{res}$ vs. RIU. (**b**) Linewidth vs. RIU. (**c**) S vs. RIU. (**d**) FOM vs. RIU.

Precise control over the depth of the PhC elements in the nm range while using high-energy particles for material processing is very challenging. During FIB milling, the overall depths of the structures can deviate from the desired values due to various parameters, such as material properties, ion-beam current and area dose [36,37,38]. This section studies the influence of PhC element depth on the performance of a nanosensor device. The sensor device was tested over three different hole depths, i.e., d=0.63a, 0.93a and 1.5a, for a standard hole shape of $R_{t}/R_{b}=0.30/0.30a$. A comparative analysis of the spectral response of the sensor device for a shallow hole with d=0.63a and a deep hole with d=1.5a is shown in Figure 10. It can be noticed that in the first case (d=0.63a) the resonant peaks are located around the wavelength range of 1570 nm, whereas in the second case (d=1.50a) the resonant peaks are located at around 1565 nm, indicating a blueshift. Additionally, the resonant speaks expand over a wider spectral range in the case of deeper holes. The performance characteristics of the sensor device for mentioned structural properties are shown in Figure 11. It can be seen in Figure 11a that the $\lambda_{res}$ redshifts linearly as a function of RIU as the value of $n_{a}$ increases for all the hole depths in cylindrical holes. However, for shallower holes of d=0.63a, the resonant peaks are located at longer wavelengths, which indicates a higher value of $n_{eff}$ in periodic structure. Figure 11b depicts the linewidth of resonant modes as a function of RIU. The trends are almost linear, ranging from 18 to 23 nm, expect a slight nonlinearity seen in the case of d=0.63a. The plot for S of the device in Figure 11c shows a linear trend around 185 nm/RIU for deeper air holes; however, it is nonlinear for shallower holes. Similarly, the variation in FOM (Figure 11d) is linear, ranging from 8 to 10 RIU^−1^ for d=0.93a and 1.50a, but it shows nonlinear behavior for shallower holes with d=0.63a.

### 4.3. Thickness of the Cladding Layer

**Figure 12:**
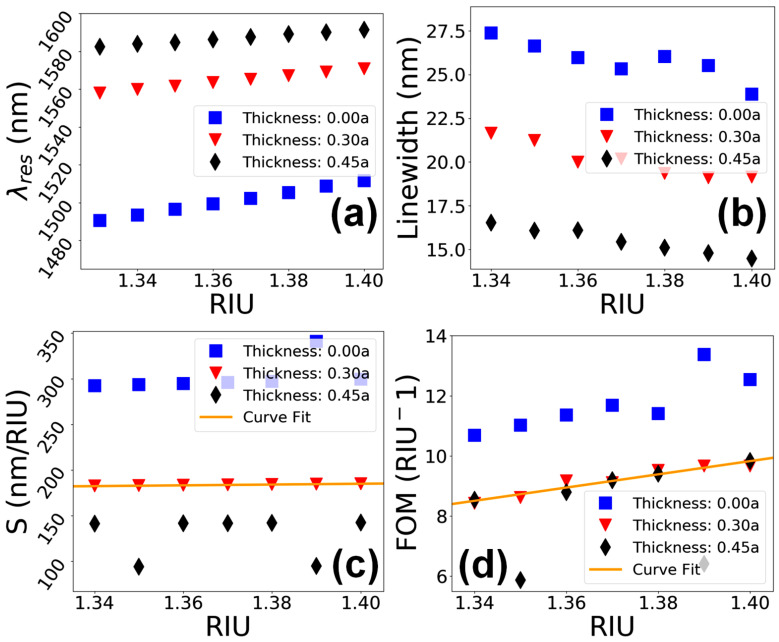
Performance evaluation of the sensor device vs. variation in the thickness of the cladding layer. (**a**) $\lambda_{res}$ vs. RIU. (**b**) Linewidth vs. RIU. (**c**) S vs. RIU. (**d**) FOM vs. RIU.

The high-energy particle beams such as FIB technologies are also corrosive to the surface of the specimen [37,38]. This corrosion mainly occurs during the scanning of the surface for selection, focusing of the beam on the working area and removal of the Pt layer by wet etching. These corrosion effects can reduce the thickness of the cladding layer, which might affect the performance of the sensor device. The optimized thickness of the cladding layer is 0.30a in the theoretical model, which corresponds to 300 nm thickness in the fabricated device. Additionally, if the thickness of the cladding layer exceeds the optimum value, it can also affect the performance of the sensor device. Therefore, the performance of the sensor was computed for three different thicknesses, i.e., c=0.00a, where the cladding layer is completely removed, optimum layer thickness of c=0.30a, and an over-deposited cladding layer of c=0.45a. The change in $\lambda_{res}$ as a function of RIU is shown in Figure 12a, which shows linear trends for all the three cladding layer thicknesses.

Moreover, the plot for linewidth in Figure 12b shows linear trends for thickness values of c=0.30a and 0.45a, reaching around 24 and 20 nm, respectively, but nonlinearity is visible when the cladding layer has a thickness of c=0.00a. Considering the S of the device in Figure 12c, the trend is perfectly linear, reaching 185 nm/RIU for an optimized cladding layer thickness of c=0.30a. However, the values of S become nonlinear when the cladding layer is non-existent, or when it becomes thicker than the optimum layer thickness. Similarly, the graphical trend for FOM (Figure 12d) is linear for c=0.30a, whereas it is non-linear for a removed or over-deposited cladding layer.

### 4.4. Sensor Performance Comparison

To find the optimal performance parameters of the proposed sensor device, the S and FOM values against all the tested structural parameters are listed in Table 2. The structural parameters include hole shape in terms of $R_{t}/R_{b}$, hole depth *d* and cladding layer thickness *c*. The performance parameters are listed in terms of S range, FOM range and differences in values of S and FOM for each tested parameter. The variations in S and FOM were calculated as the differences between the maximum and minimum values of these parameters obtained from the simulation results. The best performance parameters are listed in bold font in Table 2. The less the variation in S and FOM, the more stable the performance of the sensor device. After studying the listed variations in S and FOM parameters, it can be concluded that the device has better performance for the hole-shape range of $R_{t}/R_{b}=0.30/0.30a$ to 0.40/0.20a, a hole depth of d=0.93a and cladding thickness of c=0.30a. Moreover, for cylindrical holes with $R_{t}/R_{b}=0.30/0.30a$, the nanosensor device can give a good performance for hole depth in range of d=0.93a to 1.50a.

The performance of a sensor is highly dependent on the physical properties of the materials used and the structure of the sensor device. In general, sensors composed of metals and high-refractive-index materials offer higher values of S and FOM. This is because the optical field can attain good confinement in high-index media and the field coupled in the low-index cladding layers can only interact with the evanescent field of the resonant modes. Dielectric materials typically have a low refractive index and hence offer low-index contrast between the material layers, resulting in lower values of S and FOM. However, to achieve a cost-effective sensor design with low absorption, higher temperature tolerance and inert chemical properties, dielectric materials are always preferred. To evaluate the performance of the proposed sensor device, a comparative analysis of this work and previously reported work, mostly on dielectric materials, is shown in Table 3. The listed works include RI sensor design on 1D PhCs with dielectric materials such as Si_3_N_4_/SiO_2_ [44] and Ti_3_O_5_/SiO_2_ [45] with S values of 50 and 85 nm/RIU, respectively. Moreover, RI sensors based on Si with a higher refractive index of 3.5 are also listed. 2D PhC waveguide design [46], PhCs on SOI [47] and 2D PhC cavity in SOI [48] are also reported, having S values of 70, 94.5 and 235 nm/RIU, respectively. RI sensor designs with a ring-resonator [49] and a 2D PhC structure with a ring-slot cavity [50] are reported with S values of 200 and 160 nm/RIU, respectively. This work reports an improved value of S from 185 to 296 nm/RIU with low-index dielectric materials of SiO_2_ and Nb_2_O_5_.

## 5. Conclusions

In conclusion, a low-cost, dielectric PhC-based nanosensor was fabricated and numerically investigated in terms of its performance characteristics as an index sensor for a biological refractive range of 1.33 to 1.40. The FIB process parameters influencing the structural properties of the fabricated structures and their effects on the sensing response of the device were thoroughly investigated. For an optimum structural design of the nanosensor device with a cylindrical hole shape of $R_{t}/R_{b}=0.30a/0.30a$, the depth of the structures of d=0.93a and a symmetric waveguide design with cladding thickness of c=0.30a, the S and FOM values were found to be 185.117 nm/RIU and 9.717 RIU^−1^, respectively. However, during fabrication, keeping in mind the hardness of dielectric materials and process durations, an ion-beam current of 700 pA or above is recommended for prototyping of such a sensor device, which may result in a conical hole shape due to angular wall profile, and the depth of the air holes may also slightly deviate from the ideal value. Considering these fabrication artifacts, it is concluded that the sensor device can perform well with S in a range of 185 nm/RIU and FOM near 10 RIU^−1^ if the hole shape deviation is kept near $R_{t}/R_{b}=0.40a/0.20a$, hole depth near d=0.93a to 0.15a and cladding layer thickness near c=0.30a. Considering the size, cost-effectiveness and inert material properties of the investigated device, it can be easily integrated into the already existing fiber-optic setups and is suitable for a wide range of biosensing applications.

## Figures and Tables

**Table 1:** The structural profiles of the fabricated PhC elements measured in terms of left and right wall angles at ion-beam currents of 300, 700 and 1500 pA.

Ion-Beam Current (pA)	Left Side-Wall-Angle (Degrees)	Error	Right Side-Wall-Angle (Degrees)	Error
300	−11.61667	±0.87037	12.43333	±1.39519
700	−15.1	±1.46097	17.03333	±1.27388
1500	−13.00952	±0.63481	14.92381	±0.4589

**Table 2:** Performance comparison of nanosensor devices in terms of S and FOM with respect to variations in their structural parameters.

Hole-Shape $R_{t}/R_{b}$ (a)	Hole-Depth (a)	Cladding Thickness (a)	Sensitivity Range (nm/RIU)	FOM Range (RIU^−1^)	Variation in S (nm/RIU)	Variation in FOM (RIU^−1^)	Performance
0.30/0.30a	0.63a	0.30a	183.958–138.253	8.017–6.145	45.705	1.872	Not-linear
	0.93a		182.526–185.117	8.228–9.717	**2.592**	**1.489**	Linear/Stable
	1.50a		182.478–185.069	8.934–10.029	**2.591**	**1.095**	Linear/Stable
0.40/0.20a	0.93a	0.00a	292.543–341.085	10.682–13.3682	48.542	2.686	Not-linear
		0.30a	182.471–185.062	8.428–9.665	**2.590**	**1.237**	Linear/Stable
		0.45a	94.245–141.158	5.863–9.833	46.9126	3.9691	Not-linear
	1.50a	0.30a	218.352–266.968	8.355–11.213	48.616	2.858	Not-linear
0.50/0.10a	0.93a		136.741–184.310	6.692–10.265	47.570	3.573	Not-linear
	1.50a		252.943–296.183	10.676–13.184	43.239	2.509	Not-linear

**Table 3:** Comparative analysis of presented and previously reported works on RI sensors and their properties.

Materials	Refractive Indices	Type of Structure	Sensitivity (nm/RIU)	Reference
Si_3_N_4_, SiO_2_	2.0, 1.45	1D PhC structure	50	[44]
SiO_2_, Ti_3_O_5_	1.429, 2.285	1D PhC structure	85	[45]
Si	3.5	2D PhC waveguide on SOI	70	[46]
SOI, SiO_2_	*-*	2D PhC on SOI	94.5	[47]
SOI	3.5	2D PhC cavity in SOI	235	[48]
Si_3_N_4_	1.98	Waveguide with ring resonator	200	[49]
Si	3.48	2D PhC ring–slot cavity	160	[50]
SiO_2_, Nb_2_O_5_	1.5, 2.2	2D PhC structure	296	This Work

## Data Availability

Data availability statement not applicable.
